# “There’s just such a mismatch”: a qualitative exploration of health systems and organizational-level barriers to accessing cancer services among people experiencing structural marginalization

**DOI:** 10.1186/s12939-025-02554-8

**Published:** 2025-06-18

**Authors:** Tara C. Horrill, Jess Crawford, Scott Beck, Amber Bourgeois, Jagbir Kaur, Leah K. Lambert, Michael McKenzie, Kelli I. Stajduhar, Annette J. Browne

**Affiliations:** 1 University of Manitoba, 89 Curry Place, Winnipeg, MB R3R 2J3 Canada; 2 Nursing & Allied Health Research and Knowledge Translation, BC Cancer, Vancouver, Canada; 3 University of Victoria, Victoria, Canada; 4 Nursing & Allied Health Research and Knowledge Translation, BC Cancer, University of British Columbia, Vancouver, Canada; 5 Department of Radiation Oncology, BC Cancer, Vancouver, Canada; 6 University of British Columbia, Vancouver, Canada

**Keywords:** Cancer, Healthcare access, Health services research, Health inequities [MeSH], Healthcare disparities [MeSH], Vulnerable populations [MeSH], Structural marginalization, Social determinants of health, Equity-oriented health care

## Abstract

**Background:**

Within the context of cancer care, access to timely, high-quality care is closely correlated with better health outcomes and quality of life. Yet in Canada, research continues to show that inequities in access to cancer care persist across the cancer continuum, particularly among people experiencing structural marginalization. Although some Canadian research has explored barriers accessing cancer care, little research has explicitly focused on barriers arising from organizational and health systems contexts. Our objective was to explore barriers to accessing cancer services within the health system and organizations delivering cancer services across the cancer continuum for people experiencing structural marginalization.

**Methods:**

This study drew on critical ethnographic methods, employing a participatory, integrated knowledge translation approach. Data collection included interviews with health and social service providers (*n* = 24) and key informants (*n* = 7), interviews and focus groups with individuals with lived experience of significant health and social inequities (*n* = 29), and 40 h of observations with service providers working in clinical oncology settings. Guided by social justice, critical and intersectional theoretical perspectives, data analysis followed an interpretive descriptive approach.

**Results:**

Four interrelated themes were developed through our analysis, with the overarching thread of a ‘mismatch evident throughout: (1) the design of cancer services does not always account for social contexts and structural determinants of health; (2) discourses of operational efficiency are competing with equity-oriented care; (3) the physical spaces of cancer care matter; and (4) experiences of stigma and discrimination are incompatible with accessing cancer care. Our findings suggest that the ways in which cancer services across the continuum are designed, including the types of services available, how care activities are structured, what activities take priority, and how services are experienced, create barriers that particularly impact people experiencing structural marginalization.

**Conclusions:**

Our findings highlight the mismatches between how cancer services are currently designed and delivered, and the specific needs of people experiencing health and social inequities. These findings also point to organizations delivering cancer services as potential sites for transformation toward more equitable access to cancer care. Equity-oriented healthcare may offer a framework for service design and delivery to improve access to cancer care and experiences of care.

## Introduction

‘Accessibility’, or the ability to access health services unimpeded and without discrimination, is a key pillar of the Canadian healthcare system [1]. Health and access to healthcare is widely acknowledged as a fundamental human right, and health systems are understood as an influential determinant of health [2]. Within the context of cancer care, access to timely, high-quality care (including appropriate screening, early diagnosis, and timely intervention) is closely correlated with better health outcomes and quality of life [3–6]. Yet in Canada, research continues to show that inequities in access to cancer care persist, particularly among people experiencing structural marginalization.^1^

## Background

Cancer care services exist on a continuum, ranging from prevention/screening to early detection and diagnosis, treatment, surveillance and survivorship, and end-of-life care. In Canada, cancer care is publicly funded, organized provincially, and provided through the primary, community, and acute care systems, and dedicated cancer facilities [1, 7]. The cancer journey is acknowledged as tremendously complex, with care accessed in multiple sectors and locations (as described above), and from multiple providers (e.g., primary care providers, surgeons, oncologists). As such, cancer care has been described as ‘broken’, ‘siloed’, and ‘fractured’ [8–11]. Within Canada, the burden of cancer is growing, related to increasingly complex treatment regimens, an aging population [12], and the impacts of COVID-19 [13]. The COVID-19 pandemic saw major disruptions to the health system, including cancer prevention, early diagnosis, and treatment services, resulting in cancers that remained undetected and untreated and ultimately, the diagnosis of more advanced cancers and poorer health outcomes; these impacts are expected to be magnified among populations experiencing various forms of structural disadvantage (e.g., people with low income, racialized groups) [13]. In addition, the pandemic accelerated burnout and attrition among cancer care providers and contributed to a health human resource crisis, which continues to impact the ability of cancer services to meet patient and population needs [13].

**Fig. 1:**
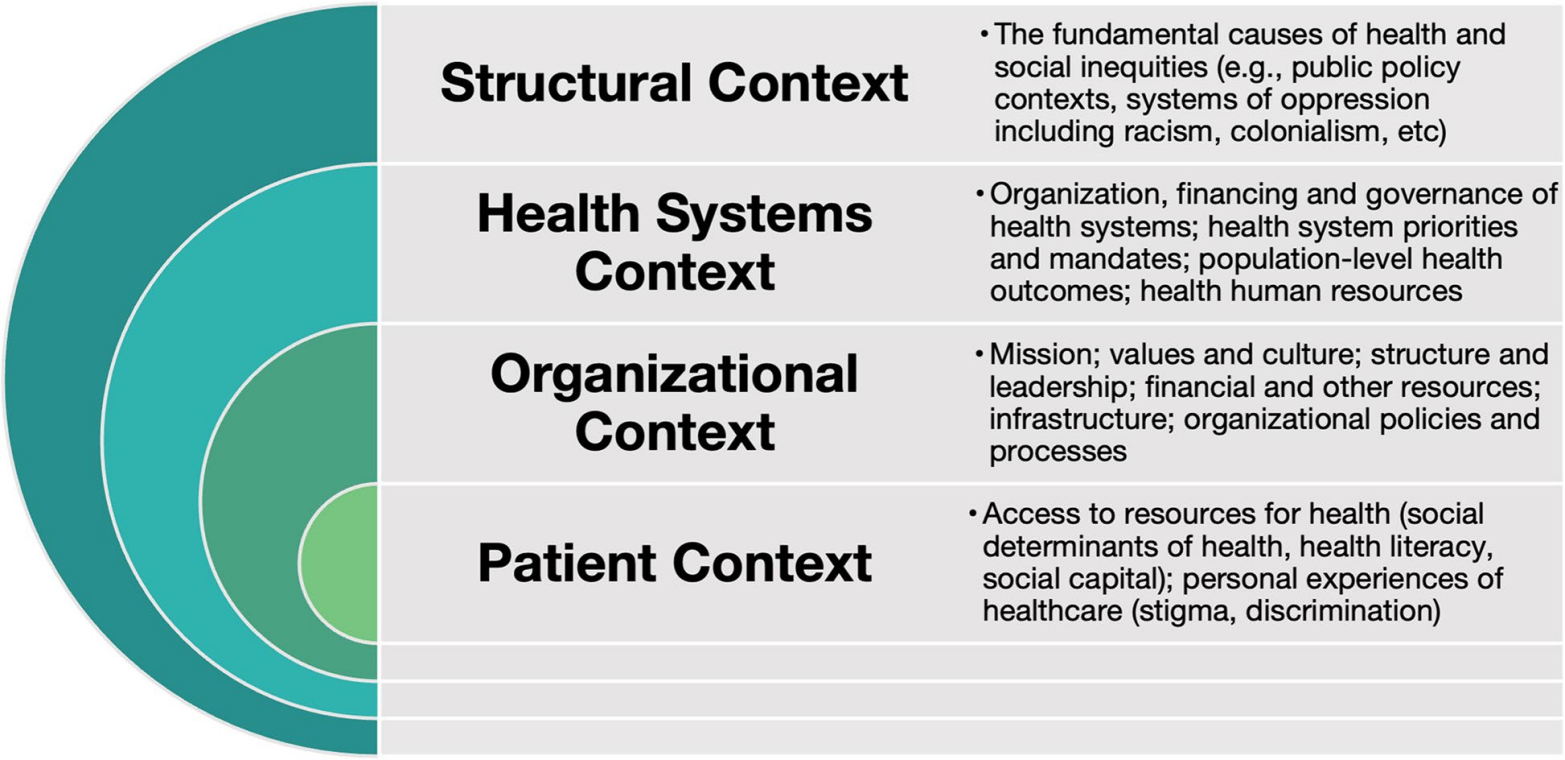
Contexts Influencing Access to Cancer Services

The confluence of an aging population, health human resource crisis, and the impacts of the COVID-19 pandemic paint a picture of health systems stretched thin, which may be contributing to and exacerbating existing cancer inequities. Evidence suggests that structurally marginalized groups in Canada are more likely to experience delayed cancer diagnosis and be diagnosed with advanced cancers, less likely to receive high quality and timely treatment, and have poorer cancer-related and quality of life outcomes [14–21]. These outcomes are not unique to Canada, and evidence suggest that similar inequities in cancer diagnosis, access to treatment, and outcomes exist in many high-income countries with publicly funded healthcare systems [22–24]. Canadian scholars investigating inequitable access to cancer care have identified a range of contexts and barriers that interact to influence access among people experiencing structural marginalization (Fig. 1). Arising from the structural context, compromised social dimensions of health, including unstable housing, food insecurity, poverty, lack of transportation and unemployment have significant impacts on access to cancer care [9, 25, 26]; meeting daily needs for survival limits capacity to engage in cancer care [11, 25]. From the perspective of patients accessing cancer care, the burden of coordinating one’s own care and a lack of trust further impact experiences of care [25, 27]. Despite the impacts of social dimensions on access to care, some evidence suggests that these needs are not consistently factored into cancer services, limiting patients’ ability to access, receive and engage in care [25, 28].

Compounding these barriers are patient experiences of racism, discrimination, and stigma when accessing cancer care [10, 25, 26]. Stigma and discrimination towards patients experiencing poverty and unstable housing or homelessness, whether real or anticipated, is noted to have significant impacts on peoples’ access to and experience of cancer care [25, 28, 29]. Gould et al. found that in some cases, people decided *not* to receive cancer treatment because of experiences of discrimination, or because of the inability of the cancer system to address social needs [28]. At the health system level, complex and siloed care, service design, and poor communication have been found to create barriers to accessing cancer care [8, 9, 11, 26, 28]. Together, this suggests that the availability of cancer care does not mean that care is equitably accessible [30]. Such barriers are not unique to the Canadian context, with evidence from high-income countries pointing to logistical, geographical, and economic barriers, negative healthcare experiences, expectations that patients coordinate and monitor their own care and compromised social dimensions of health all impacting access to and experiences of cancer care [22, 23, 31–35].

Organizations delivering cancer services, themselves nested within the health system, also play important roles in addressing inequities at the point of care and facilitating access to timely, high-quality cancer care through organization-specific approaches to care and partnering with community-based organizations [7]. Some Canadian research has pointed to challenges in the ways cancer services are designed and delivered that create barriers to accessing cancer care, yet little extant research has explicitly focused on barriers arising from organizational and health systems contexts. These are important to understand given that there are growing calls to prioritize health equity in cancer care [36] and that the cancer care sector is increasingly recognized as a critical site for advancing equity [7].

Equity-oriented healthcare has been proposed as one approach to designing and delivering healthcare services that are purposefully responsive to health and social inequities, by aiming to reduce the impacts of structural violence (e.g., poverty) and various forms of oppression (e.g. racism) [37, 38]. Transformative equity-oriented healthcare is grounded in three key dimensions embedded across organizations: a) trauma- and violence-informed care; b) culturally safe and anti-racist care; and c) harm reduction philosophies and non-stigmatizing care [36, 39, 40]. The effectiveness of these approaches to equity-oriented healthcare have been studied in Canadian primary care and emergency departments settings [37, 39–41] and have been identified as relevant and applicable for implementation in the cancer care sector [36].

## Methods

In this paper, we discuss findings from a study that explored the integration of equity-oriented care approaches into the design and delivery of cancer services in one Western Canadian province. Our specific objective for this analysis was to explore barriers to accessing cancer services within the health system and organizations delivering cancer services across the cancer continuum for people experiencing structural marginalization. We drew on critical ethnographic and interpretive description methods and employed a participatory, integrated knowledge translation (iKT) approach, in which healthcare providers and decision makers were a part of the research team and involved in all aspects of the study. We also partnered with a community-based primary care clinic whose mandate is to provide health and social services to people who are structurally marginalized in a large urban area.

### Theoretical perspectives

Our study was grounded in social justice aims, drawing on critical, intersectional theoretical perspectives [42, 43]. Critical and intersectional perspectives posit that social identities (i.e., age, ethnocultural identity, class, gender, sexuality, disability) and institutions (e.g., social, political, economic, and cultural institutions) interact to construct systems of oppression [44, 45]. Intersectional theories have been applied in health research to highlight how one’s positionality (the various identities one has) results in different experiences of oppression and power, and subsequently, differing access to care [46, 47]. With the aim of developing practice knowledge to redress a particular injustice or inequity, critical theoretical perspectives focused our attention on how complex social, political, and power dynamics impact the design and delivery of healthcare services, the ability of healthcare providers to meet the needs of patients, and the capacity of people to access healthcare [42–44]. Taking this critical intersectional lens offered an opportunity to highlight less obvious barriers to accessing cancer services in order to identify opportunities to redress health and healthcare inequities, and to build on the current strengths of the system [48, 49].

### Data collection

This study took place in two large urban areas of one Western Canadian province. Data were collected between June 2022 and December 2023. Purposive and nominated sampling techniques were used to recruit three groups of participants: (a) health and social service providers (e.g., nurses, nurse practitioners, physicians) working in an oncology setting or in a community-based setting providing care and services to structurally marginalized people (‘service providers’); (b) people with lived/living experience of structural marginalization (e.g., poverty, unstable housing, food insecurity) (‘patients’), and (c) organizational, clinical, or policy leaders working across the cancer continuum (‘key informants’). Service providers and key informants were invited to participate by email through the community-based primary care clinic we partnered with and our professional networks, and through study information posted online. Participating service providers also referred other potential participants through snowball or nominated sampling. To invite patients/people with lived experience, we posted invitations to participate in the facility operated by our partner organization. Community-based health and social service providers in our partner organization also provided study information and focus group information to patients who met the inclusion criteria.

We initially planned to focus data collection with patients on their past experiences related to obtaining care for a suspected or confirmed diagnosis of cancer, however, as we began data collection with service providers, and engaged in conversations with our partner organization, it became apparent that many ‘patients’ experiencing structural marginalization may never receive an official cancer diagnosis or receive treatment as a result of the compounding barriers to accessing cancer services. Moreover, a cancer diagnosis in general can be a painful and traumatic process and may be layered onto experiences of diagnostic care and treatment that require repeated exposure to ‘mainstream’ healthcare services typically not designed in ways that meet the needs of people experiencing structural marginalization. As a result, we shifted to focus on patient perspectives of how cancer services across the continuum could be made more accessible, equity-oriented, and welcoming as a way of drawing on their intimate knowledge and experiences of cancer services without asking participants to specifically recount and relive those experiences.

#### Focus groups and semi-structured interviews

Semi-structured interviews were conducted with service providers and key informants in person or via Zoom. Semi-structured virtual interviews and in-person focus groups with individuals with lived experience of health and social inequities were conducted between October and December 2023. Service providers (who participated outside of their regular work responsibilities) and patient participants were offered a cash honorarium in recognition of their time and expertise. Interviews were transcribed verbatim by a professional transcriptionist. Detailed fieldnotes were recorded by hand during focus group discussions and transcribed afterwards.

#### Observational field notes

Observations were conducted by TH in two urban cancer centers between October 2022 and October 2023 and employed an equity-oriented approach [50]. The first author spent approximately 40 h observing service providers working in clinical oncology settings (e.g., outpatient oncology clinics, radiation treatment clinics), yielding important contextual information about the physical spaces of care, interactions between providers and patients, workflows, the types of tasks providers engage in, the impact of processes (who and how the work is done), formal and informal policies impacting how work is done, and interactions among various types of providers. Although observations included service providers’ work within their respective organizations (e.g., oncologists, registered nurses), the primary focus was on the organizational contexts, including the factors and complexities of service delivery that limit or foster service provider and organizational capacity for equity-oriented care. Field notes were used to record observations (e.g., physical environment), reflexive (e.g., personal impressions of the researcher), analytic (e.g., how the observation/s may relate to other data collected, broader literature, or theoretical framework), and methodological (e.g., noting questions to ask in future interviews) notes. Interview transcripts, focus group notes, and observational field notes were imported into Dedoose qualitative analysis software (www.dedoose.com) for analysis.

### Data analysis

Our approach to data analysis was informed by interpretive description [51]. Data were analyzed inductively and iteratively, beginning with repeated readings of interview transcripts and field notes. An initial coding framework was developed and refined with codes added and collapsed as data analysis progressed, and applied across the data set [52]. As patterns became evident, coded data were clustered into categories. In keeping with the thrust of interpretive description towards development of contextual and practice-relevant findings, in the later stages of analysis, we shifted to a more abstract, conceptual analysis of thematic findings, with the aim of articulating the implications of these findings for health service design and healthcare delivery [51, 53, 54]. Analysis was led by TH and JC in consultation with AJB and KS; evolving themes were brought to the larger team for critique and discussion as the analysis progressed. Given that team members brought varying social identities, and disciplinary, methodological, and substantive knowledge to the analysis, processes of reflexivity were embedded into these discussions (e.g., explicitly or implicitly discussing why we gravitated towards a particular interpretive understanding of the data). In the last stages of our analysis, we hosted six discussion sessions in which we invited service providers affiliated with our partner organization, patient participants, and community members. This served as a way to engage in conversations regarding study findings, report back to the communities, and consider how we might move forward in future research and community engagement (more fully described in a forthcoming publication). All study participants were also provided with a summary of findings and were offered multiple opportunities to provide feedback. Throughout analysis, an audit trail of analytic and interpretive decisions was maintained.

### Participant characteristics

Participants included 24 service providers, 7 key informants, and 29 patients experiencing structural marginalization. Service provider (SP) participants included participants from a range of disciplines and roles, of which over half were professional nursing roles (RNs and NPs). More than half of SP participants (58%) worked in community-based and/or primary care settings. Key informants (KI) worked in a variety of leadership roles, and included participants working at the regional, provincial and national levels. Patient participants identified primarily as women (73%). More than half self-identified as Indigenous (59%); this is likely because of the significant impacts of health and social inequities on Indigenous peoples within Canada, and the large population of Indigenous peoples in the geographical areas from which we recruited study participants. Patient participants (PT) experienced multiple forms of structural disadvantage (see Table 3), with 31% indicating it was very difficult and 45% indicating it was somewhat difficult to live on current household income. Participant characteristics are summarized in Tables 1,2 and 3.

**Table 1:** Service Provider Participants

Participant Characteristics	N = 24 (%)
*Employment Role*	
Nurse (RN)	7 (29%)
Nurse Practitioner	6 (25%)
Physician	3 (13%)
Outreach/Support Worker	2 (8%)
Allied Health Professional	2 (8%)
Manager/Coordinator	2 (8%)
Other	2 (8%)
*Setting*	
Community-based or primary care	14 (58%)
Oncology care	9 (38%)
*Highest Level of Education*	
Graduate degree	0 (0%)
Undergraduate degree or college diploma	21 (88%)
Some university or college	3 (13%)
Highschool diploma or GED	0 (0%)
*Years in Current Role*	
< 1	0 (0%)
1–5	13 (54%)
6–10	3 (13%)
11–15	5 (13%)
16–20	2 (8%)
20 +	1 (4%)
*Years in Health or Social Services Field*	
1–5	2 (8%)
6–10	8 (33%)
11–15	2 (8%)
16–20	7 (29%)
20 +	5 (21%)

**Table 2:** Key Informant Participants

Participant Characteristics	N = 7 (%)
*Employment Role*	
Executive or operational leader	2 (29%)
Clinical leader	3 (42%)
Policy maker	2 (29%)
*Setting*	
Community-based or primary care	1 (14%)
Oncology care	6 (86%)
*Highest Level of Education*	
Graduate degree	6 (86%)
University degree	1 (14%)
*Years in Current Role*	
< 1	0 (0%)
1–5	4 (57%)
6–10	2 (29%)
11–15	0 (0%)
16–20	0 (0%)
20 +	1 (14%)
*Years in Health or Social Services Field*	
< 1	0 (0%)
1–5	0 (0%)
6–10	1 (14%)
11–15	0 (0%)
16–20	2 (29%)
20 +	4 (57%)

**Table 3:** Patient Participants Experiencing Structural Marginalization

Participant Characteristics*	N = 29 (%)
*Age*	
Average Age	56
Age range	27–77
*Gender*	
Woman	21 (73%)
Transwoman	1 (3%)
Man	5 (17%)
Not specified	2 (7%)
*Indigenous Status*	
Self-identified as Indigenous	17 (59%)
Self-identified as non-Indigenous	10 (34%)
Prefer not to answer or not specified	2 (7%)
*Highest Level of Education*	
Graduate degree	1 (3%)
University degree or college diploma	3 (10%)
Some university or college	3 (10%)
Highschool degree or GED	7 (24%)
Some high school or middle school	10 (34%)
Completed elementary school	2 (7%)
Don’t know or not specified	3 (10%)
*Current Living Situation*	
Private apartment/condo/house	7 (24%)
Public, social or supportive housing	13 (45%)
Couch surfing	1 (3%)
Shelter	3 (11%)
On the street	1 (3%)
Single-room occupancy hotel	2 (7%)
Prefer not to answer or not specified	2 (7%)
*Current Work Status*	
Employed full time (20 + hours/week)	1 (3%)
Employed part time (< 20 h/week)	3 (10%)
Seasonally employed	1 (3%)
Unemployed	10 (34%)
Retired	3 (10%)
Services in exchange for food/housing	1 (3%)
Student	2 (7%)
Other	5 (17%)
Prefer not to answer or not specified	3 (10%)
*Sources of Income*	
Social assistance	4 (14%)
Disability benefits	16 (55%)
Pension	6 (21%)
Prefer not to answer or not specified	3 (10%)
*Difficulty Living on Current Household Income*	
Very difficult	9 (31%)
Somewhat difficult	13 (45%)
Neutral	1 (3%)
Somewhat easy	4 (14%)
Very easy	0 (0%)
Prefer not to answer or not specified	2 (7%)

^*^Options where n = 0 are not reported

### Findings: ‘There’s just such a mismatch’

**Fig. 2:**
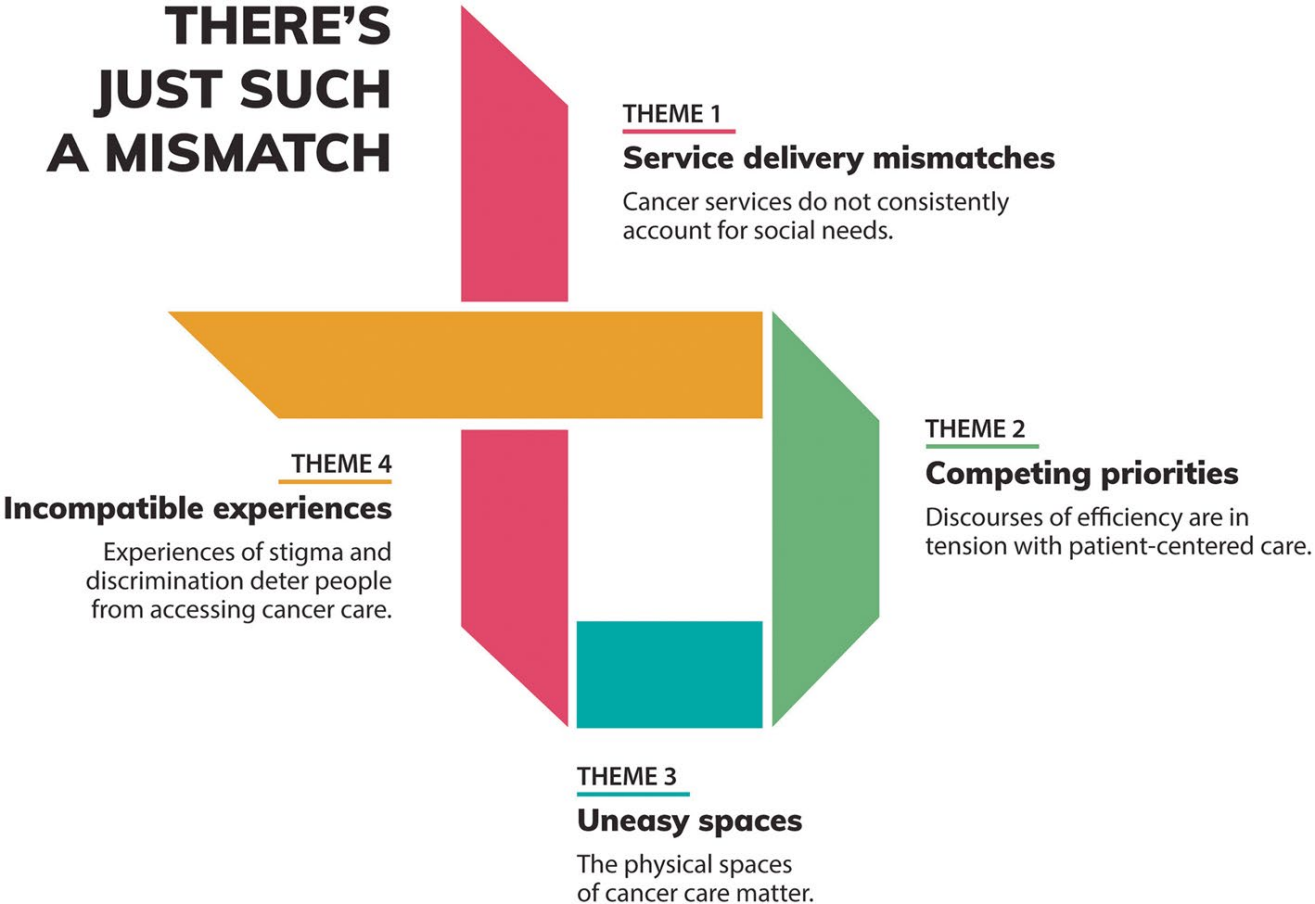
Thematic diagram of barriers to cancer care

Our findings suggest that the ways in which cancer services across the continuum are designed, including the types of services available, how care activities are structured, what activities take priority, and how services are experienced, create barriers that particularly impact people experiencing structural marginalization. The overarching theme of our analysis was that of a ‘mismatch’. Four interrelated themes were developed through our analysis, with the overarching thread of a ‘mismatch evident throughout: (1) the design of cancer services does not always account for social contexts and structural determinants of health; (2) discourses of operational efficiency are competing with equity-oriented care; (3) the physical spaces of cancer care matter; and (4) experiences of stigma and discrimination must be mitigated to promote equitable with access to cancer care (Fig. 2).

### Service delivery mismatches: challenges attending to social needs

People experiencing structural marginalization often have significant unmet social needs that need to be mitigated and sometimes addressed before they can meaningfully access cancer services. At the same, cancer services (and the Canadian health system more broadly) have historically not been delivered in ways that effectively recognize social needs as integral to health. One of the most significant barriers described by all groups of participants was how cancer services were delivered in ways that made it challenging and sometimes impossible to consider these social needs (such as housing, for example), and peoples’ experiences of trauma, stigma, or discrimination. As a result, a pattern of mismatches between the care provided and the specific needs of patients experiencing structural marginalization was evident. Repeatedly, participants described how designated cancer services tended to prioritize biomedical and physiological needs, often at the expense of other aspects of patients’ lives, rendering social contexts and needs less visible. One participant reflected on their experiences in this way:

“The reality of cancer care is that sometimes the care that you receive is dictated by the expression of a certain protein on the surface of a particular kind of cell…I respect that that’s a mainstay of what we do. But the problem is because we work in an environment that is so dictated by what is happening on a cellular level, we have created the perfect conditions to neglect everything else.” (KI01)

The privileging of biomedical and physiological needs was evident in whether and how social needs were assessed and addressed. Observational field notes and interviews documented that, although there were existing processes for assessing social needs (for example, intake assessment forms with questions related to housing, finances and social supports), these were inconsistently conducted (and if conducted, only at first intake), were lengthy, and often completed by patients themselves, creating possibilities for less robust assessment and significant social needs to remain hidden. Moreover, when psychosocial issues were identified in the context of cancer treatment services, they were most often addressed through referrals to patient counselling services; some SP participants perceived that these providers were not well integrated into the care team, and that the referral mechanisms created a lack of care continuity. In contrast, multiple SP participants emphasized the need for a model of care in which an interdisciplinary team works collaboratively to address the full range of patient needs, including some aspects of care coordination. While community-based service providers expressed tremendous frustration at the seeming limited capacity within specialized cancer services to attend to social needs, some oncology clinicians also expressed frustration, and at times distress, in feeling as though there was little they could do to meaningfully address these needs.

#### Service delivery mismatches: disconnections between cancer services and primary care

In this study, we found that many people experiencing health and social inequities had strong links to health and social service providers in the community (most likely a reflection of our partnership with a community-based primary care clinic), who were able to develop trusting relationships and offer continuity of care. These providers functioned to coordinate various aspects of patients’ health and social care, including complex schedules for cancer treatment, particularly for patients who experienced unstable housing and relatedly, lack of access to a telephone, internet, or a fixed mailing address. Yet repeatedly, we heard from these community-based service providers about communication disconnects – experiences in which critical communication about patient appointments or other aspects of care did not reach the patient *or* the community-based service provider working with the patient to support them. One community-based service provider elaborated:

“Most of our clients don’t have cell phones… So, it would be great if they could contact us to notify any appointments. If appointment generation was not contingent on having initial contact with clients. So, like, not calling them on the phone and yes, they confirm that this date works, you know? Like *can I not be that person for my clients*? Particularly when they consent to that. Which they do, if they don’t have a cell phone or an address. We always have those conversations.” (SP23)

Communication disconnects seemed to represent one of the most salient challenges from the perspective of community-based service providers, and in several instances, persisted despite explicit instructions from patients and providers, along with signed consent, that communication should be directed to the community-based provider instead of the patient. Communication disconnects often resulted in missed and cancelled appointments, and circumstances in which cancer screening, diagnostic tests, and treatment were delayed or skipped, with potentially detrimental impacts for people with lived experiences of inequities. Moreover, the disconnect highlights a missed opportunity to harness the resources and trusting relationships with patients that exist within community-based settings.

“We have this one medical oncologist at [cancer institution], their office will not tell us – for some reason you have a signed consent and everything, we coordinate all her appointments, she has two appointments a week. I’m her care coordinator, and they refused to contact me with her appointment information for the specialist, they will only let her know. But she is right now quite chaotic and having some memory issues and other competing needs and doesn’t tell us and then misses the appointment. And so, I literally have sent them consents from her saying please call my social worker with the information. And she has missed the last three appointments because they won’t.” (SP06)

We also heard from some oncology-based service providers, and observed in fieldwork, that an absence of standardized mechanisms within electronic or paper medical records to document the primary point of contact when it is not the patient themselves. For example, during fieldwork, SP25 demonstrated that to indicate an alternate contact in the electronic medical record, an electronic ‘sticky note’ could be added to the chart, yet these are often reportedly difficult for other team members to find, even if actively looking.

#### Service delivery mismatches: ‘Fitting into the box’ and meeting health system expectations

Health systems broadly, and the cancer care system specifically, are designed to meet the needs of people who can adhere to the often firm timelines and schedules designed to move patients through systems. These were sometimes described by service providers as being “typical” or “ideal” patients (e.g., SP18). Yet people experiencing structural marginalization often have significant social and physical needs that may not be directly related to their cancer diagnosis, which may remain unaddressed in the current cancer care context:

“I think where it doesn’t work well is that because we are used to a certain way of functioning that if there’s anything additional that may be requiring a little bit more resources or different types of resources, then it’s harder to navigate how to access those. Even though I think the intention is there, but it’s just the structure is not” (SP12).

Adding to the fundamental mismatch between the current health system and the needs of people experiencing structural marginalization were the unwritten and often unacknowledged expectations of the healthcare system and entrenched hierarchies of power, in which health and social service providers are often in positions of power in relation those accessing care. One participant described the expectations of the health system:

“I mean where there’s expectations of behaviors, there’s a norm. There’s a reciprocal relationship established and expected, that you would be told to sit down, and you will wait for your appointment, and you will thank them and then you will go in when you’re called; all that… and you’re capable of following the directions to whatever the treatment that is on offer.” (SP11)

The above quote highlights a challenge identified by multiple service providers who felt that patients had to ‘fit into the box’ to meet the expectations of the cancer care system by arriving to appointments on time and being prepared to wait patiently. One service provider further articulated how such expectations created specific challenges:

“For our folks who use substances, one real barrier for them – that’s an equity issue for sure – is that our medical system is often set up, where you have to be at an appointment, at a certain time, at a certain location – so you have to have money to get there, have a means of transportation to get there and then you have to sit, then you have to wait – and often you have to wait for a very long time – and so folks might anticipate those and bring a book or bring a tablet or whatever it is that they’re going to do to occupy themselves with the waiting time. But if you have a very active substance use disorder and you start to go into withdrawal, you’re not going to be able to wait.” (SP02)

Several participants (patients and service providers) perceived that when patients were not able to fit neatly into the box and meet the expectations of the health system, they were unable to access cancer services. For example, multiple community-based service providers described experiences of attempting to support patients through a colonoscopy as part of colon cancer screening, and the expectation that patients will have access to housing that includes a private or semi-private washroom to complete the bowel preparation required. These service providers articulated how they were rarely able to navigate this process for their patients whose housing did not have appropriate washroom facilities in the absence of a connection with a sympathetic physician who was willing to admit the patient to hospital in advance of the colonoscopy to facilitate the bowel preparation.

### Competing priorities: discourses of operational efficiency in tension with equity-oriented care

In the current context of wait lists to access cancer services, health human resource and financial constraints, organizations delivering cancer services are faced with the realities of ensuring operational efficiency. Discourses of operational efficiency were evident in how several SP and KI participants described the context of cancer service delivery organizations: “we’re in…the walls of a one size fits all, ‘we’ve got to be efficient and get people through’ mentality, [and it] just doesn’t serve people well” (KI04). This presented particular challenges when providing care to people experiencing structural marginalization as the drive towards meeting the needs of the organization through the efficient use of resources (personnel, time, space, funding) appeared to compete with the need to provide flexible, tailored care (hallmarks of an equity-oriented approach). Many service provider participants articulated a desire for an equity-oriented approach, yet this was in tension with the realities of their clinical practice contexts, including the urgent need to address a backlog of patients who are suffering while waiting for care. For example: “…you have to maintain staff and flow and basically provide care to the majority, but you need to have people that are also super flexible that can meet people where they’re at” (SP20). Yet this push towards maintaining or improving operational efficiency filtered through decision making in ways that tend to disproportionately impact people already experiencing multiple forms of disadvantage; this was most evident through inflexibility in appointment systems, and a perceived lack of agency among service providers, which we explore further below.

#### Competing priorities: inflexibility of cancer appointments

Inflexibility in how cancer services are delivered was identified as a major challenge significantly impacting access to cancer services for people experiencing structural marginalization. This was exemplified primarily through inflexible and sometimes punitive appointment policies, more colloquially referred to as the ‘3 strikes’ rule. One participant explains: “…we have a policy about patients that miss multiple appointments. And the policy – and I was against this policy – is like, you know, if they missed three appointments, we cancelled them and handed it off to the oncologist and say, you know, you need to make sure this patient is going to show up” (SP21). Such policies, whether formal or informal, exemplify how discourses have their effects through language and institutional processes. Many participants, particularly service providers working in community-based settings, described their immense frustration with such policies, and the detrimental impacts on the people they work closely with:

“…there’s a lot of reasons why they can’t make it to the clinic on time, that might not necessarily be their fault. And even if it was, our job is to provide them with healthcare. So are we actually serving them by saying – this very patriarchal system of – ‘your appointment was at this time. And if you don’t get here at this time, then too bad so sad for you.’” (SP17)

While recognizing how inflexibility disproportionately impacts those who experience barriers to accessing cancer care, some participants also recognized that maintaining flexibility (and the associated potential for increased use of resources) within the current context of ongoing health human resource issues and post pandemic impacts, is immensely challenging. At the individual provider level, “when you don’t have as much staff and everybody’s working at or above capacity, it’s hard to be flexible” (SP18). Another participant described the broader challenges:

“We are just, kind of on the smallest part of riding the wave of the baby boomer explosion of cancer that’s going to happen. And the impact on [low] staffing from COVID and responding to the higher rates of cancer from an aging population means that we are facing unprecedented challenges in being able to keep up with the demand. We are experiencing huge wait lists to get in for treatment and clinicians are struggling to keep up with the demands of seeing as many people as possible. There is this thing, you know, that if you take longer to see someone else, or if someone does not show for their appointment that you may have said ‘no’ to someone else who is waiting. So, there is this whole ethical dilemma how should we be providing care. Is it to the population or the person in front of you? How are we able to make workarounds in an already very strained system, and how do you do that as a provider- knowing that you’re one person. It is causing a lot of people to burn out.” (SP20)

Despite the inherent challenges in maintaining some flexibility in care delivery, this was identified as a key factor in equitable access to cancer services (particularly for structurally marginalized groups), perhaps pointing to the need for a degree of autonomy among healthcare providers in deciding how to best tailor care to patient needs.

#### Competing priorities: lack of healthcare provider agency

Operational efficiency discourses seemed to have the impact of limiting innovation and agency at the clinician level. One key informant repeatedly expressed that there was little room for creative problem solving, stating “you can’t really innovate” (KI04); another stated “innovation is an easy thing to say, but most building structures or organization people have really little space for innovation or time to even sort of test out alternative models, per se” (KI02). Several SP participants described examples of their attempts to make small changes within their clinical practice to better meet the needs of patients experiencing various inequities which they perceived to be difficult. For example, SP20 described a desire to adapt an intake assessment form to reflect locally relevant and equity specific concerns, but that this was stifled: “and so people, when they hear, oh, you can’t do that, it puts the brakes on a lot of innovation. So there’s a lack of innovation going on, I think, because of, you know, basically, like, having to go through multiple layers of leadership.” Several SP and KI participants alluded to how this organizational context was reflective of the broader system in which changes within healthcare organizations are themselves hampered by layers of bureaucracy within healthcare systems, health authorities, and ultimately, governmental ministries of health.

One specific mechanism that limited the agency of oncology service providers was the use of care pathways and standardized workflows. While such pathways are implemented with the goals of maximizing patient safety and treatment efficacy, minimizing risk, and ensuring efficiency this opened the potential for care that was less equity-oriented, with fewer opportunities for clinicians to make use of their clinical wisdom. For example, one key informant described at length how specific clinical protocols and care pathways are primarily biomedically driven. They described that in the context of cancer care for people who are structurally marginalized, however, assessment of the social determinants of health and the social contexts within which individual patients live is a key factor to their uptake and adherence to cancer treatment and care and may present specific ‘risks’ and needs that are essential to address. Flexibility in care approaches was paramount according to some SP and KI participants, as patients, because of their social and structural constraints, may not be able to tolerate the rigidity of some care pathways. KI02 provided an example of how the flexibility to facilitate those clinical responses does not exist within the current system, and as a result, providers are not able to tailor care in ways that are equity oriented:

“We have follow-up phone calls that are happening to certain patients because it’s on [a treatment] protocol, but many people wouldn’t know that the value and impact of [those calls] from a patient perspective is actually very minimal. So we had a knee-jerk response because there was a biomedically driven decision around ‘there’s a risk associated here’… But we’re not appreciating risk or experience of the impact of social determinants and how we’re care planning around a patient in the way that we easily could.” (KI02)

Despite their distress and perceived lack of agency, multiple service provider participants’ accounts of their own clinical work demonstrated small acts of resistance to efficiency discourses. For example, in the earlier quote by SP21, she stated that she “was against” the 3-strikes policy, and did not enforce this in her own practice; SP20 described a similar experience: “I kept having to rebook him. I had rebooked him five times to get his treatment. So his treatment wasn’t on time and a lot of other people would have been like, three strikes and you’re out. You know?”.

### Uneasy spaces: the physical spaces of cancer care matter

Patient and community-based service provider participants repeatedly emphasized the importance of attending to the physical spaces in which cancer services are delivered. These spaces were described by focus group participants as feeling depressing, colonial, not welcoming, cold, lonely, and uncomfortable. One participant recalled her work as an outreach worker, accompanying and supporting a patient in attending their cancer treatment appointments:

“He was like, I feel like I’m going into jail, what’s going on. And it’s not that it looks like jail, I think it’s more that it’s just such a sterile environment and it’s just not – you know … generic and sterile. It’s not some place you want to go every day for however many months…because it being institutionalized, it’s not such a welcoming space. […] As long as you don’t have paint color that looks like the same colors as jail, no blues, no creams, just not the same colors as jail. I mean, it’s also being mindful that a lot of our people have been in residential care in some shape or form so those clinical settings are not so welcoming and can be rather triggering for some of them” (SP04).

Other participants emphasized the profound impact of experiences of institutionalization, noting that these experiences can deter people from accessing healthcare: “…we have all kinds of people that have been in foster care and residential care facilities at young ages because of what’s gone on in their lives and they do not want any feeling of institutionalization” (SP05).

Fieldnotes indicate that some of the clinical spaces we observed within organizations delivering cancer services had the look and feel of a hospital – wide hallways lined with hand railings and medical equipment, fluorescent lighting and linoleum flooring, staff dressed in scrubs and white coats, larger patient rooms partitioned with curtains and smaller exam rooms lining the hallways. Yet there were also attempts to make patient waiting areas comfortable and welcoming, through the use of artwork and varying paint color on the walls, water coolers, and televisions. SP20 described that prior to the COVID-19 pandemic, volunteers were often present in patient waiting areas serving coffee, drinks, and snacks, in addition to volunteers who played instruments or brought therapy dogs. However, these services were put on hold at the beginning of the pandemic, and at the time of data collection, had not been reinstated. SP20 points out that these types of services can address many basic needs (including food and hydration), while also making patients and their caregivers feel calmer.

Finally, observations noted that patient check-in desks (reception desks) often included a glass or clear plastic barrier between the staff member and public space, with only a small opening to pass documents through. Several participants, including service providers and patient participants, noted frequent lineups at these desks. Fieldnotes documented prominently displayed signage at many of these desks and in other public spaces of a ‘zero-tolerance’ policy: “Violence, foul language and abusive behavior are not acceptable. Verbal threats or acts of violence will not be tolerated and may result in removal from this facility and/or prosecution.” While individually, these aspects of the clinical space may seem innocuous, together, they contributed to perceptions of being unwelcomed in these clinical spaces.

### Incompatible experiences: stigma and discrimination are incompatible with accessing cancer care

Layered onto experiences of feeling unwelcomed, negative healthcare interactions further deterred people from accessing cancer care. Experiences of stigma, discrimination and judgment in cancer care compound previous experiences within health and social care systems more broadly: “…the way they are treated in hospitals, that’s what it all comes down to… A lot of them just don’t want to go to doctors because they’ve had such horrible experiences” (SP10). Several community-based service provider participants commented that their patients would rather die than pursue treatment for their diagnosed cancer, as a direct result of previous negative experiences with the healthcare system, relating to histories of mental health and substance use challenges.

Multiple community-based service providers described witnessing patients experiencing stigma, particularly related to histories of substance use, when accompanying patients to cancer appointments:

“I’ve had the experience of bringing a client [to see] an oncologist and the oncologist is privy to some information about their personal history that has nothing to do with their cancer health. And it’s like, I’m going to focus on that, instead of focusing on your cancer care, and it does definitely happen, that very blatant judgment and stigma” (SP14).

Moreover, two participants described experiences in which patients they were supporting were told that they could not receive cancer treatment unless they abstained from using substances entirely: “the whole idea that someone would have to stop using drugs in order to access some kind of care…that’s going to make that person feel extremely stigmatized and judged and not comfortable, and probably not want to go back” (SP08).

In focus groups with patient participants, personal experiences with a cancer diagnosis or having lost a loved one to cancer were nearly ubiquitous. More striking was the undercurrent of distrust towards the institution of healthcare more broadly, represented in participants’ questions about whether they would be offered cancer treatment based on evidence (the most effective treatment) or cost (the least expensive treatment). Several other patient participants described experiences in which they felt judged and stigmatized because of their housing and/or economic situations. For example, PT22 shared her experience of having attended an appointment the previous day at the cancer center, and tearfully shared how she perceived the physician to be “rude”, “judgmental” and “abrasive” during the appointment; the participant felt this was a result of her current living situation, which found her living in a shelter. Together, our findings suggest that stigma, discrimination and judgment from healthcare providers constitute a significant barrier to accessing cancer services. Although such barriers occur at the individual level, they do so within organizational and health systems contexts that at worst, permits this type of behavior, and at best, have not taken sufficient meaningful action to actively counteract it.

## Discussion

This study identified multiple barriers to accessing cancer services arising from organizational and health systems contexts. Notably, our findings reiterate the complexity of accessing cancer services, and the intersecting nature of barriers to accessing care. Our analysis demonstrates how barriers can originate from health service design, underlying discourses influencing care delivery, physical spaces of care, and patient experiences of each of these, resulting in fundamental mismatches in care.

The findings discussed in this paper suggest that cancer services are designed to privilege physiological cancer care, with broader health and social needs of patients often being less visible. Although it may appear that some providers are challenged to adequately meet non-biological needs, this is likely a function of the dominance of the biomedical model of care, which emphasizes biological causes and treatment of disease, and tends to focus on individuals outside of their social context, noted elsewhere as a barrier to equitable cancer care [55]. Critiques of the dominance of biomedicine have problematized it’s dualistic and reductionistic nature [56]. World renown social epidemiologist Nancy Krieger has written extensively on the problematic dominance of biomedicine within the field of cancer health equity globally [57]. While biomedicine has evolved over the last several decades (primarily as a result of the evidence-based medicine movement), it remains reductionistic, with care “organized around body parts instead of treating the whole patient” (44 p641). Biomedical dominance is also reinforced within and perpetuated by organizational contexts [58]. For example, Mescuoto et al., conducting research in the Australian healthcare context, describe how macro-organizational policies such as maximum appointment times create pressures on clinicians who must then prioritize their care, often limiting care to biologically-oriented care [59]. This suggests that organizations can also play a role in shifting towards a model of care that is equity-oriented, thereby expanding capacity to assess and address social determinants of health.

In addition to a fundamental lack of attention to patients’ social contexts, scholars note that the dominance of biomedicine also results in siloing of care and expectations that patients self-manage aspects of their care [60]; this phenomenon was also identified in a scoping review of cancer inequities in high-income countries with publicly-funded healthcare services, suggesting it is not an issue that is specific to the Canadian context [33]. While the complexity and siloing of cancer services has been described elsewhere as a notable barrier to accessing cancer care [8, 10, 11, 61], Truant notes that this siloing also “limits collaborative opportunities for addressing structural constraints that perpetuate health inequities” (46 p216). In our study, this was evident in the communication disconnects between services delivered in primary/community-based settings and services delivered in specialized cancer institutions in ways that were potentially or actually detrimental to patients’ access to care. In particular, communication and coordination of cancer-related appointments was directed towards patients who were not equipped to receive that communication, rather than collaborating with primary and community-based service providers who were well positioned to work with patients in navigating a complex cancer journey. This may be reflective of subtle and rising expectations of ‘self-management’, which is common in cancer service delivery contexts, but particularly problematic in the context of the complexities of cancer service delivery documented in Canada and the US [25, 31, 32, 61]. Yet this also has additional implications for people experiencing structural marginalization who often have few material resources, the least power, and are at risk of not receiving the care they need [62]. However, this also points towards an opportunity for cancer service delivery organizations to harness the resources and relationships already existing to deliver equitable care.

The tensions created between care that is centered around the needs of the organization and the healthcare system in which it is embedded, and care that is centered around the unique needs of patients experiencing structural marginalization represent another form of mismatch. Healthcare reforms in Canada over the past several decades have seen a shift towards the corporatization of healthcare, including the application of business-like practices to a public good and human right [63]. Several scholars have noted that a push towards health service design and delivery that prioritizes efficient processing (the movement of patients through the system as quickly and cheaply as possible) has closely accompanied this shift [49, 60, 64, 65]. Dubbed by some scholars as the ‘McDonaldization’ of healthcare [64], ‘LEAN oncology’, and a ‘culture of efficiency’ [55], Truant argues that “the impact of corporatization on clinical systems encourages efficient movement of individuals through the cancer care system, possibly at the expense of other needs, such as addressing psychosocial issues” (46 p217). Other scholars have articulated how standardized care has been prioritized within the push towards evidence-based medicine, leaving little room for the individualization of care that is needed to ensure equitable access [67]. This is particularly concerning for populations experiencing structural marginalization who are most in need of contextually-tailored care [62], and in which psychosocial needs, when unaddressed, prevent people from receiving life-prolonging, life-saving, and symptom reducing treatments [8, 25].

While we recognize that organizational discourses of efficiency are embedded within the larger context of corporatization of healthcare in Canada, the impacts on clinicians and care is significant: clinicians are left with the responsibility of ‘rationing’ services to make the most of limited resources (e.g., limiting time spent on care coordination) and enforcing efficiency related policies (e.g., the ‘three strikes’ rule). In their research in Emergency Departments in urban areas of Western Canada, Slemon et al. found that both operational efficiency and equity-promoting discourses were operating at the same time, but in different ways [68]. Whereas discourses of efficiency and ‘flow’ were used to uphold dominant institutional power structures, equity discourses were invoked as a subversive strategy for “taking up practices that do not directly align with dominant institutional processes and structures” (52 p10). Similarly, our findings suggest that clinicians invoke equity-promoting discourses through small acts of resistance (e.g. rebooking patients more than 3 times, booking patients for longer than normal appointments). However, despite these acts of resistance, or perhaps because of them, the tensions described in our findings may be creating conditions of moral distress among clinicians.

Moral distress is conceptualized as a personal reaction to situations in which health and social service providers believe they know what the ‘right’ or ethical thing to do is, but are unable to do it [69]. In the current Canadian cancer service delivery setting, evidence suggests that efficiency and ‘business’ mindsets have created pressures for clinicians to provide less holistic care in exchange for productivity, thereby contributing to moral distress [66]. Scholars have also articulated that while patient-specific situations can result in moral distress, it is often organizational, systemic, and structural factors that are most distressing – where providers have the least influence yet witness some of the most devastating impacts [49, 62, 66, 69]. We observed several oncology service providers working to be as flexible as possible yet constrained in providing care that was truly equity-oriented because they continue to experience significant workload constraints, and work within systems that are not equitably designed. Such notable gaps between what healthcare providers *know is required* to deliver equitable and high-quality care, and what they are *able to provide* suggests that attention to the organizational and health systems contexts in which providers are delivering care is a critically important step to move towards equity-oriented cancer care.

Although little research has focused specifically on experiences of stigma, trauma and discrimination in the context of cancer care, and substance use stigma in particular, these have all been identified as barriers to cancer care within Canada and beyond [8, 10, 25]; structural and systemic racism have been well documented as root causes of cancer inequities in the US [70]. In line with extant research, our findings suggest that stigma and discrimination act both to deter patients from accessing care and to compound other systemic and structural barriers. We emphasize that it is unlikely that oncology care providers are intentionally drawing on discriminatory and stigmatizing views in their care delivery, and the vast majority of healthcare providers are committed to providing the highest quality of care possible. However, these realities do not negate the devastating impacts of patient experiences; attention to power dynamics and anti-oppressive training is critically important in healthcare provider education and continuing professional development. Finally, our data suggests that some participants questioned the role of stigma in treatment and care decision making. Although this specific issue was beyond the scope of our study, we recognize that treatment decision making in the context of cancer and people experiencing structural marginalization is tremendously complex; while substance use and other forms of stigma may be playing a role in sustaining or deepening existing inequities, additional research is needed to understand the nuances of provider decision making processes and impacts on equitable access to cancer care.

### Strengths & Limitations

Findings from this analysis should be considered within the context of several strengths and limitations. First, given the inclusion criteria for patient participants (people with lived/living experience of structural marginalization) and the extensive evidence that people from structurally marginalized groups often experience challenges when accessing care in Canadian health care systems, it is not surprising that participants in this study described numerous examples of negative experiences; our findings may also be limited by our recruitment of patient participants from one particular underserved community. However, these experiences did provide context to our analysis of barriers. Second, our analysis of organizational-level barriers to accessing cancer care may have been impacted by the larger proportion of service provider participants who worked in community-based settings than in oncology-based settings, who may have had less experiential knowledge of the cancer care system. However, these service providers brought in-depth knowledge of the social and healthcare contexts experienced by patients experiencing structural marginalization, and experiential knowledge of supporting these patients to access various forms of cancer care. Additionally, our analysis was strengthened through the use of multiple methods of data collection representing diverse perspectives and social locations.

## Conclusion

Despite growing body of evidence suggesting persistent inequities in access to cancer care, and that social and structural determinants of health serve as barriers to accessing cancer care among people and groups experiencing structural marginalization, fewer studies have explored the reasons for these inequities. In particular, few studies have explored barriers to accessing cancer services arising from organizational and health systems contexts. Our findings highlight the mismatches between how cancer services are currently designed and delivered, and the specific needs of people experiencing health and social inequities. These findings also point to organizations delivering cancer services as potential sites for transformation towards equitable access to cancer care, and ultimately, equitable cancer outcomes. Yet to date, the potential for transformation has remained largely unrealized, and strategies and interventions to advance health equity in cancer care continue to be ‘proposed’ or ‘recommended’ rather than implemented [7]. Meaningful and tangible actions are needed that address organizational and health systems contexts. Equity-oriented healthcare may offer a framework for service design and delivery to improve access to cancer care and experiences of care, by offering flexible, tailored care and helping to foster a sense of safety, comfort and respect [36]. Moreover, advancing health equity in cancer care will require strategic, coordinated, and collaborative approaches across the health system and beyond.

## Data Availability

Because of privacy and ethical considerations, these data are not publicly available. Inquiries regarding the original dataset can be directed to TH.

## Footnotes

1 Structural marginalization refers to the process of marginalization and exclusion resulting from political, economic, social and historical structures, and the uneven distribution of resources, power and opportunities. Structural marginalization shifts the focus from understanding marginalization as a result of individual characteristics to a focus on the impacts of social structures.
